# Sexual life and dysfunction after maternal morbidity: a systematic review

**DOI:** 10.1186/s12884-015-0742-6

**Published:** 2015-11-23

**Authors:** Carla B. Andreucci, Jamile C. Bussadori, Rodolfo C. Pacagnella, Doris Chou, Veronique Filippi, Lale Say, Jose G. Cecatti, on behalf of the Brazilian COMMAG Study Group and the WHO Maternal Morbidity Working Group

**Affiliations:** Department of Obstetrics and Gynecology, University of Campinas, Campinas, Brazil; Federal University of Sao Carlos, Sao Carlos, Brazil; Reproductive Health Research unit, World Health Organization, Geneva, Switzerland; London School of Hygiene and Tropical Medicine, University of London, London, England UK

**Keywords:** Systematic review, Maternal morbidity, Maternal near miss, Sexual dysfunction, Dyspareunia

## Abstract

**Background:**

Because there is a lack of knowledge on the long-term consequences of maternal morbidity/near miss episodes on women´s sexual life and function we conducted a systematic review with the purpose of identifying the available evidence on any sexual impairment associated with complications from pregnancy and childbirth.

**Methods:**

Systematic review on aspects of women sexual life after any maternal morbidity and/or maternal near miss, during different time periods after delivery. The search was carried out until May 22^nd^, 2015 including studies published from 1995 to 2015. No language or study design restrictions were applied. Maternal morbidity as exposure was split into general or severe/near miss. Female sexual outcomes evaluated were dyspareunia, Female Sexual Function Index (FSFI) scores and time to resume sexual activity after childbirth. Qualitative syntheses for outcomes were provided whenever possible.

**Results:**

A total of 2,573 studies were initially identified, and 14 were included for analysis after standard selection procedures for systematic review. General morbidity was mainly related to major perineal injury (3^rd^ or 4^th^ degree laceration, 12 studies). A clear pattern for severity evaluation of maternal morbidity could not be distinguished, unless when a maternal near miss concept was used. Women experiencing maternal morbidity had more frequently dyspareunia and resumed sexual activity later, when compared to women without morbidity. There were no differences in FSFI scores between groups. Meta-analysis could not be performed, since included studies were too heterogeneous regarding study design, evaluation of exposure and/or outcome and time span.

**Conclusion:**

Investigation of long-term repercussions on women’s sexual life aspects after maternal morbidity has been scarcely performed, however indicating worse outcomes for those experiencing morbidity. Further standardized evaluation of these conditions among maternal morbidity survivors may provide relevant information for clinical follow-up and reproductive planning for women.

## Background

Maternal mortality and severe morbidity have been identified by the World Health Organization (WHO) as key indicators for the evaluation of women’s health worldwide [1, 2]. Little is known about the long-term consequences of severe maternal morbidity, since the majority of studies on this subject evaluates women not longer than six weeks after delivery [3]. After birth, several disorders may occur: post-traumatic stress disorder, postpartum depression, physical and emotional disabilities, and sexual dysfunction [4–7]. Women who suffered complications during pregnancy and childbirth may present clinical and psychological disorders that may last for long time [8]. Thus, these conditions may lead to deterioration of quality of life and adverse effects on both mother and child.

Several factors may influence and affect the health and quality of life of women who had episodes of Severe Maternal Morbidity (SMM) or Maternal Near Miss (MNM). MNM is a condition defined by the WHO as “a woman who nearly died but survived a complication that occurred during pregnancy, childbirth or within 42 days of termination of pregnancy”, while maternal morbidity is “any condition that is attributed to or aggravated by pregnancy and childbirth that has a negative impact on the woman’s wellbeing” [1, 9].

Sexual health is a state of physical, mental and social well-being in relation to sexuality, and the World Health Organization (WHO) also considers its quality as a health indicator [10]. In this context, sexual dysfunction in fact may be considered a consequence of maternal morbidity [11]. However, there are not many studies addressing this issue.

General medical disorders and treatments may interfere with sexual motivation, desire, subjective arousal and excitement, orgasm, pleasure, and freedom from pain [7, 12–14]. Any increased delay in resuming sexual intercourse after childbirth, even after taking in account cultural and ethnic variations, could be considered as an important issue on female sexual response, since it is caused by an altered hormone level condition. Hormone levels interfere with desire and arousal, and therefore can impact on sexual function [15].

The subjectivity and complexity of sexual function led to the development of several instruments for its evaluation, the following are the main known and used ones. The Female Sexual Function Index (FSFI) is a questionnaire to be applied to evaluate the female sexual response fields (phases or components of sexual response): sexual desire, sexual arousal, vaginal lubrication, orgasm, sexual satisfaction and pain [16]. Intimacy Relationship Scale (IRS) was designed to assess sexuality among couples after childbirth [17]. Sexual Function Short Form Questionnaire (PISQ-12) is a self-administered instrument to evaluate sexual function in women with pelvic organ prolapse and/or urinary incontinence [18]. The Sexual Function Questionnaire (SFQ-) was proposed as a tool for investigation and diagnosis of female sexual dysfunction adding to the former domains the couple relationship (including sexual) [19]. The Maudsley Marital Questionnaire (MMQ) was validated in the early 80s in order to address sexual relationship among couples living together [20].

Therefore the research question for this systematic review is whether there is available evidence on any sexual impairment associated with complications from pregnancy and childbirth. A review of scientific literature on any kind of sexual health impairment associated with maternal morbidity, severe maternal morbidity and/or maternal near miss may improve the current knowledge on the topic. Assuming that the more severe the morbidity, the more serious the impact on sexual function, possibly introducing it in the follow up of women who suffered such conditions will enable the development of further prospective studies to gather more powerful evidence on this relationship. Thus, a better understanding on the long-term consequences of maternal morbidity on women’s sexual quality of life may provide support for future research and action.

## Methods

This is a systematic review on aspects of the sexual life among women that experienced any maternal morbidity and/or maternal near miss, during different time periods after delivery. The searches for publications were carried out until May 22^nd^, 2015. Studies included were published from 1995 to 2015, with no language or study design restrictions, since they were identified in the specific databases selected.

Any study design providing prevalence or incidence rates for any sexual function aspect (outcome) and maternal morbidity condition (exposure) in any population was included for assessment. A minimum sample size was not required for study inclusion. Those could include cross-sectional, case–control and cohort studies. Maternal morbidity could be diagnosed by clinical, management or laboratory criteria or even self-reported. Short and long-term morbidity and *sequelae* were included. For morbidity related to integrity of perineum, we only considered those reported as third and/or fourth degree laceration as a major injury. Episiotomy was considered as an intervention and not an obstetric complication.

Exclusion criteria for eligibility of studies for the systematic review:

- Studies with no original data or where no dates for data collection periods are provided;
- Theoretical or review articles;
- Studies specifically looking at the consequences of emergency Cesarean section;
- Reports referring to data collected before 1995.
- Qualitative studies.

The electronic databases that were searched from the year 1995 through 2015 were: PubMed, EMBASE and SciELO. Some pilot test searches were performed before selecting the current set of databases, which showed to be wide and effective enough for identifying the studies of interest. This search was independently performed by two reviewers and, therefore, double-checked, with discrepancies solved by a third senior reviewer. Search strategies were customised for each electronic database according to their individual subject headings, syntax and searching structure. The main key words used were “mothers”, “maternal”, “maternal morbidity”, “severe maternal morbidity”, “maternal near miss”, “obstetric complication”, “pregnancy complication”, “obstetric morbidity”, “puerperium”, “postpartum”, “after childbirth”, “sexual function”, “sexual functioning”, “sexual health”, “sexual dysfunction”, “sexual activity”, “sexuality”, “sexual behaviour”, “dyspareunia” and “Female Sexual Function Index”. We applied the MeSH Terms tool when applicable and used no filter to select the studies from those databases.

**Fig. 1:**
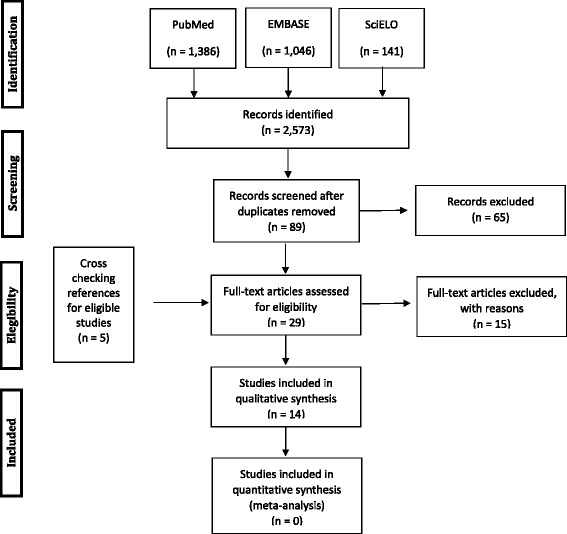
PRISMA Flow Diagram for Female Sexual Dysfunction after Obstetric Complications

Search strategy and the flow of selection for studies are shown in Fig. 1. All citations identified by the electronic search strategies in each database were initially evaluated according to the screening form based first on their titles and secondly on their abstracts. All abstracts selected were available. Studies that did not meet the criteria regarding title and/or abstract were considered irrelevant. Therefore they were discarded. The same occurred with duplicates identified in more than one database.

Additionally, checking the list of references of these possible eligible studies for inclusion, reviewers could select and analyse further publications. Full texts of the remaining studies were obtained. The studies were evaluated on their quality using the following parameters: sampling (targeted sample population cleared defined), description of the population, period of follow-up reported, completeness of data for the whole sample, and clear description of diagnostic procedure and definition of outcome. Those rated as inadequate on a global assessment of these parameters according to the reviewers were excluded.

A screening form was developed for this systematic review, in order to address the research question, i.e., which outcomes have been already studied after exposure to any complication during pregnancy, childbirth and/or extended postpartum period. The full text reports were evaluated according to this screening form, which consists of: a) Mapping for the definition of female sexual altered response (outcome). The evaluation of sexual function/dysfunction considered results from validated questionnaires (FSFI/IRS/PISQ-12/FSQ-/MMQ-S); b) Description of time interval to resume sexual activity after delivery; c) The database search for articles that correlate morbidity during pregnancy, childbirth and postpartum period (exposure), sexual function defined above and different timing on postpartum sexual abstinence; d) Correlation of described sexual life aspects with maternal morbidity and/or maternal near miss.

The studies excluded at this stage, as well as the reasons for their exclusion, are listed in Table 1.

**Table 1:** Studies excluded with their respective reasons for exclusion

Author/Year/Location	Title	Reasons for exclusion
1. Saurell-Cubizolles MJ et al., 2000. France/Italy	Women´s health after childbirth: a longitudinal study in France and Italy.	No complications assessed.
2. Fornell EU et al., 2005. Sweden	Obstetric anal sphincter injury ten years after: subjective and objective long term effects.	No complications assessed.
3. Williams A et al., 2007. UK	The prevalence of enduring postnatal perineal morbidity and its relationship to perineal trauma.	No information on the degree of perineal laceration.
4. Leeman LM et al., 2007. USA	Do Unsutured Second-Degree Perineal Lacerations Affect Postpartum Functional Outcomes?	Definition of major perineal trauma included 2^nd^ degree laceration.
5. Rogers RG et al., 2009. USA	Does spontaneous genital tract trauma impact postpartum sexual function?	Definition of major perineal trauma included 2^nd^ degree laceration.
6. Lal M et al., 2011. UK	Does post-caesarean dyspareunia reflect sexual malfunction, pelvic floor and perineal dysfunction?	Definition of perineal trauma included 2^nd^degree laceration and/or episiotomy.
7. Fauconnier A, et al., 2012. France	Late post-partum dyspareunia: Does delivery play a role?	No information on the degree of perineal laceration.
8. Crane AK et al., 2013. USA	Evaluation of Pelvic Floor Symptoms and Sexual Function in Primiparous Women Who Underwent Operative Vaginal Delivery Versus Cesarean Delivery for Second-Stage Arrest.	No complications assessed.
9. McDonald EA & SJ Brown, 2013. Australia	Does method of birth make a difference to when women resume sex after childbirth?	No information on the degree of perineal laceration.
10. Lurie S et al., 2013. Israel	Sexual function after childbirth by the mode of delivery: a prospective study.	No complications assessed.
11. Rikard-Bell J et al., 2014. Australia	Perineal outcome and the risk of pelvic floor dysfunction: A cohort study of primiparous women.	No information on the degree of perineal laceration.
12. Adanikin AI et al., 2014. Nigeria	Resumption of intercourse after childbirth in southwest Nigeria.	No information on the degree of perineal laceration.
13. Song M et al., 2014. Japan	Association Between Sexual Health and Delivery Mode.	No information on the degree of perineal laceration.
14. McDonald EA et al., 2015. Australia	Dyspareunia and childbirth: a prospective cohort study.	No information on the degree of perineal laceration.
15. Faisal-Cury A et al., 2015. Brazil	The Relationship Between Mode of Delivery and Sexual Health Outcomes after Childbirth.	Episiotomy and perineal laceration evaluated as the same outcome.

Data from the remaining relevant studies were independently extracted by two reviewers, using a pilot-tested data-extraction form or framework especially prepared for this review (including authors, year, study design, what and how outcomes were assessed, period of data collection, population and results of outcomes), and they were then compared. A third senior reviewer was available for discussion and decision when data extracted by the two first reviewers differed. Attempts to directly contact authors to obtain additional information or clarification were performed.

Following the initial screening, included studies were appraised in order to map the differences and similarities in their characteristics and decide whether a meta-analysis was recommended on the basis of low heterogeneity; or whether a qualitative synthesis of the available results would be more appropriate in case of high heterogeneity. The qualitative synthesis included description of study design, population, sample size and association between exposure and outcomes. The procedures used for this study and report followed those recommended by the PRISMA statement [21].

## Results

Our search through the selected databases is summarized in Fig. 1. The electronic search identified a total of 2,573 studies using the developed search strategy. After title assessment, 89 studies were evaluated through their abstracts and 65 were excluded at this point. The full text of 24 studies were analysed, in addition to 5 other papers identified by checking reference lists of included papers. The 15 studies that did not fulfil the inclusion and exclusion criteria were excluded and they are listed in Table 1, along with the reasons for exclusion [22–36]. As a result, 14 studies were included in this systematic review (Tables 2 and 3). Among them, 12 studies focus on perineal laceration as maternal morbidity exposure and 2 studies evaluated women after episodes of what authors defined as any type of severe maternal morbidity and/or maternal near miss.

**Table 2:** Description of included studies with perineum injury morbidity as exposure

Publication Authors Year of publication	Type of study Outcome Assessment	Period of data collection Location	Participants	Outcomes
1. Women´s sexual health after childbirth. Barret G et al., 2000 [37]	Cross sectional Sexual problems (vaginal dryness, painful penetration, dyspareunia, vaginal tightness, vaginal looseness, bleeding/irritation after sex and loss of sexual desire) Mailed questionnaires.	1^st^ July to 31 December 1997 London, UK	484 postpartum primiparous women. Evaluation at 3 and 6 months after delivery.	Dyspareunia at 3 months after birth: Minor perineal laceration: 55 % (115/209) 3^rd^ degree laceration: 64 % (7/11) Dyspareunia at 6 months after birth: Minor perineal laceration: 26 % (62/235) 3^rd^ degree laceration: 33 % (4/12)
2. Postpartum sexual functioning and its relationship to perineal trauma: A retrospective cohort study of primiparous women. Signorello LB et al., 2001 [38]	Retrospective cohort 1. Time to resumption of sexual intercourse 2. Dyspareunia Sexual satisfaction Sexual sensation Likelihood of achieving orgasm. Mailed questionnaires.	From August 1, 1996, and February 8, 1997. Boston, Massachusetts, USA.	626 postpartum women (6 months) Group 1, n = 211 (intact perineum or first-degree perineal tear) Group 3, n = 68 (third- or fourth-degree perineal trauma)	1. Resumption of sexual activity: In 7–8 weeks Group 1: 30.8 % (61/211) Group 3: 35.0 % (21/68) In 6 months: Group 1: 94.8 % (199/210) Group 3: 92.5 % (62/67) Time to resume in weeks (mean and SD): Group 1: 7.1 (3.3) Group 3: 9.3 (3.5)*p < 0.001* 2.Pain (dyspareunia) at first intercourse: Group 1: 57.8 % (115/211) Group 3: 77.4 % (48/68) (*p* < 0 .001). 3 months postpartum: Group 1: 32.8 % (60/211) Group 3: 51.4 % (35/68) (*p* trend < 0 .001) 6 months postpartum: Group 1: 18.6 % (37/211) Group 3: 26.7 % (16/68) 3. Sexual satisfaction, sexual sensation and achievement of orgasm.
3. Which factors determine the sexual function 1 year after childbirth? van Brummen et al., 2006 [42]	Prospective cohort MMQ-S scale Self-applied questionnaires at pregnancy (12 and 36w), and at 3 and 6 months postpartum.	From January 2002 to July 2003. Netherlands	377 women evaluated at 3 periods (pregnancy, 3 and 5 months postpartum)	Sexual activity 1 year after childbirth (resumption): YES: Intact/1^st^/2^nd^ degree laceration: 46,3 % 3^rd^/4^th^ degree laceration: 56,5 % NO: Intact/1^st^/2^nd^ degree laceration: 53,7 % 3^rd^/4^th^ degree laceration: 43,5 % MMQ-S questionnaire: No differences among groups (NS)
4. Women’s health 18 years after rupture of the anal sphincter during childbirth: II. Urinary incontinence, sexual function, and physical and mental health. Otero et al., 2006 [44]	Retrospective cohort. FSFI Mailed questionnaire	18 years after childbirth that occurred between January 1st, 1982, and December 31th, 1983. Geneva, Switzerland	453 women. Exposed: 230 (women who had had anal sphincter injury during childbirth) Not exposed: 223 (randomised postpartum women not exposed to the injury and with the same parity)	FSFI <25: NS Anal Injury: 39/230 No injury: 38/223 RR 1,0 (CI 95 % 0,8-1,2 *p =* 0.75)
5. Long Term effects of anal sphincter rupture during vaginal delivery: faecal incontinence and sexual complaints. Mous et al., 2007	Retrospective cohort study Sexual complaints (including dyspareunia)	2005 assessment of postpartum women who gave birth between 1995 and 1996. Rotterdam, Netherlands	Total included in 2005: 119 cases (sphincter injury) 90 controls (no injury)	Dyspareunia: Sphincter injury: 29 % (35/119) Controls: 13 % (12/90)
6. Women's dyspareunia after childbirth: a case study in a hospital in Acapulco, Mexico. Solana-Arellano, et al., 2008 [40]	Case control study. Dyspareunia.	From October 2005 to January 2006 Acapulco, Mexico.	Cases: 152 postpartum women who resumed sexual activity and referred pain or bleeding during intercourse. Controls: 152 postpartum women who resumed sexual activity and not referred the same symptoms. 2–6 months after childbirth. 27 women had perineal laceration.	Perineal laceration: (27/152) 17.8 % in cases with dyspareunia (23/152) 15.1 % in controls without dyspareunia
7. Sexual Function 6 Months After First Delivery. Brubaker et al., 2008 [43]	Prospective cohort Resumption of sexual activity at 6 months Sexual Function Short Form Questionnaire (PISQ-12)	September 2002 and September 2004 USA	536 postpartum primiparous women at term, 459 sexually active. Sphincter tear: 198 Vaginal controls: 200 Cesarean controls: 61 Interviewed at 6 weeks and 6 months after childbirth.	Resumption of sexual activity at 6 months: Sphincter tear: 88 % (171/198) Vaginal controls: 93.9 % (13/200) Cesarean controls: 85.9 % (51/61) *p* = 0.028 Mean PISQ-12 scores at 6 months: 39 ± 4 *p* = 0.92 NS
8. Tears in the Vagina, Perineum, Sphincter Ani, and Rectum and First Sexual Intercourse after Childbirth: A Nationwide Follow-up. Rådestad I et al., 2008 [48]	Prospective cohort Resumption of sexual activity Mailed questionnaire	Between 1999 and 2000 Sweden	2,134 postpartum women at 2 months and 1 year after childbirth (vaginal delivery). Tears in sphincter ani/rectum: 59	Resumption of sexual activity: Tears in sphincter: ≥3 Months: 49.2 % ≤3 Months: 23,3 % RR = 2.1 (CI 1.6–2.8) *p < 0.001* No tears in sphincter: ≥3 Months: 23.3 % ≤3 Months: 76.7 % RR = 1 Difference between groups: 25.8 % (49.2 %–23.3 %) NNH: 3.9 (CI 95 % 2.6-7.7) Tears in sphincter: ≥6 Months: 13.6 % ≤6 Months: 86.4 % RR = 1.8 (CI 0.9–3.5) *p = 0.130* No tears in sphincter: ≥6 Months: 7.6 % ≤6 Months: 92.4 % RR = 1 Difference between groups: 6.0 % (13.6 %–7.6 %) NNH: 16.8 (CI 95 % 6.8-35.2) At 12 Months: 4 % NO sexual activity (NS)
9. Sexual Function in Women 3 Days and 6 Weeks After Childbirth: A Prospective Longitudinal Study Using the Taiwan Version of the Female Sexual Function. Chang, et al., 2010 [45]	Prospective longitudinal FSFI	From November 2007 to April 2009 Taipei, Taiwan	356 postpartum women 199 vaginal delivery (no intact perineum)	FSFI Day3: 1^st^/2^nd^ degree laceration (182/199) LSM = 7.0 3^rd^ or 4^th^ degree: (17/199) LSM = 7.1 *p* = 0.984 (NS) FSFI Week 6: 1^st^/2^nd^ degree (158/172) LSM = 13.6 3^rd^ or 4^th^ degree (14/172 ) LSM = 15.1 *p* = 0.7 (NS) Sexual activity score Day 3: 1^st^/2^nd^ degree laceration (182/199) LSM = 15.0 3^rd^ or 4^th^ degree: (17/199) LSM = 14.1 *p* = 0.8548 (NS) Sexual activity score Week 6: 1^st^/2^nd^ degree laceration (182/199) LSM = 18.8 3^rd^ or 4^th^ degree: (17/199) LSM = 20.9 *p* = 0.7193 (NS) Satisfaction score Day 3: 1^st^/2^nd^ degree laceration (182/199) LSM = 51.7 3^rd^ or 4^th^ degree: (17/199) LSM = 56.0 *p* = 0.6691 (NS) Satisfaction score Week 6: 1^st^/2^nd^ degree laceration (182/199) LSM = 151.1 3^rd^ or 4^th^ degree: (17/199) LSM = 166.5 *p* = 0.4723 (NS) Desire score Day 3: 1^st^/2^nd^ degree laceration (182/199) LSM = 6.2 3^rd^ or 4^th^ degree: (17/199) LSM = 31.4 *p* = 0.1703 (NS) Desire score Week 6: 1^st^/2^nd^ degree laceration (182/199) LSM = 271,0 3^rd^ or 4^th^ degree: (17/199) LSM = 233.8 *p* = 0.3056 (NS)
10. Obstetric anal sphincter injury in the UK and its effect on bowel, bladder and sexual function. Marsh F et al., 2010	Prospective cohort Resumption of sexual activity Dyspareunia	From 2004 to 2009 Leeds, UK	435 postpartum women with obstetric anal injury (up to 3 Months after delivery)	Resumption of sexual activity: 57 % (134/235) Dyspareunia: 32 % (75/235)
11. Pelvic floor dysfunction 6 years post-anal sphincter tear at the time of vaginal delivery. Baud et al., 2011 [46]	Case–control study FSFI	From 1996 to 2006 Lausanne, Switzerland	Cases: 66 postpartum women with anal injury (1,5 % from 13,213) Controls: 192 without anal injury. Up to 6 years postpartum.	FSFI ≤ 25 (severe dysfunction): NS Data not available: contact authors. FSFI total scores: Cases: 26.1 + 6.8 Controls: 27.3 + 5.9 *p* = 0.185
12. The effects of mode delivery on postpartum sexual function: a prospective study. De Souza et al., 2015 [47]	Prospective cohort FSFI Self-applied questionnaire	From January 2010 to July 2011 Melbourne, Australia	391 women interviewed during pregnancy and after 6 and 12 months after childbirth Completed 3 interviews: 9/264 (4.8 %) women with 3^rd^ degree laceration 82/264 (44.1 %) with minor perineal injury	FSFI score (means): Pregnancy: 24.22 6 months: 22.79 12 months: 25.06 No differences associated to type of laceration Arousal domain, means (maximum 6): Pregnancy: 3.46 6 months: 3.44 12 months: 3.97 *p = 0.007* 12 months highest score related to perineal injury p = 0.019 Orgasm domain, means (maximum 6): Pregnancy: 4.23 6 months: 4.20 12 months: 4.66 p = 0.026 6 months/12 months = 0.015 No interaction over time due to perineum status (p = 0.108) FSFI scores < 25 not showed

**Table 3:** Description of included studies with severe maternal morbidity/maternal near miss as exposure

Publication Authors Year of publication	Type of study Outcome assessment	Period of data collection Location	Participants	Outcomes
13. Postnatal morbidity after childbirth and severe obstetric morbidity. Waterstone M, 2003	Prospective cohort Resumption of sexual activity Problems with sexual function Problems with sexual function related to EPD score	Data collected between 1st March 1997 and 28th February 1998. South East Thames Region, UK	329 exposed (cases) 1,330 not exposed (controls) Total = 1,670	Resumption of sexual activity: Cases: ≤6 w = 43 % (123/329) 7–12 w = 43 % (125/329) >12 w = 14 % (40/329) Controls: ≤6 w = 56 % (709/1,330) 7–12 w = 35 % (437/1,330) >12 w = 9 % (118/1,330) χ^2^ = 17.66 *p < 0.001*. Problems with sexual function: Cases: 34.1 % (77) Controls: 18.7 % (240) (95 % CI for difference 8.9 % to 22.0 %; χ^2^ = 27.5, df = 1, *p* < 0.001). EPDS score & problems with sexual function: >13 = 27.1 % (95/351) 10 – 12 = 28.5 % (74/260) <10 = 17.3 % (182/1052) (χ ^2 =^22.9, df = 2, *p* < 0.001).
14. Women’s sexual health and contraceptive needs after a severe obstetric complication (“near-miss”): a cohort study in Burkina Faso. Ganaba R et al., 2010	Prospective cohort Resumption of sexual activity Dyspareunia	Data collected between December 2004 and March 2005. Burkina Faso, Africa	1,014 postpartum women diagnosed with near miss 3, 6 and 12 months after delivery Perinatal death/abortion: 120 Induced abortion: 18 Live births: 199 Uncomplicated births: 677	Resumption of sexual activity: At 3 months Per. death/miscarriage: 64/114 (56.1 %) Induced abortion: 8/18 (44 %) Live birth: 44/178 (24.7 %) Uncomplicated birth: 186/657 (28.3 %) At 6 months Per. death/miscarriage: 94/111 (84.7 %) Induced abortion: 11/15 (73.3 %) Live birth: 88/164 (53.7 %) Uncomplicated birth: 388/640 (60.6 %) At 12 months Per. death/miscarriage: 96/103 (93.2 %) Induced abortion: 12/15 (80 %) Live birth: 106/155 (68.4 %) Uncomplicated birth: 444/597 (74.4 %) Dyspareunia: NS At 3 months Per. death/miscarriage: 15/64 (23.4 %): Induced abortion: 1/8 (12.5 %) Live birth: 12/44 (27.3 %) Uncomplicated birth: 58/156 (31.2 %) At 6 months Per. death/miscarriage: 10/86 (11.6 %) Induced abortion: 2/10 (20 %) Live birth: 13/82 (15.9 %) Uncomplicated birth: 51/375 (13.6 %) At 12 months Per. death/miscarriage: 1/8 (12.5 %) Induced abortion: 1/1 (100 %) Live birth: 3/23 (13.0 %) Uncomplicated birth: 6/88 (6.8 %)

Table 2 shows included studies addressing general morbidity. The outcomes assessed regarding aspects of sexual life and/or dysfunction were dyspareunia, sexual function scores assessed by several female sexual evaluation questionnaires, any sexual complaint or problem, depressive symptoms, and time to resumption of sexual activity after childbirth. Particularly, sexual function was evaluated through 3 different instruments (MMQ, FSFI and PISQ-12), and also through specific domains or phases of female sexual response.

Accordingly, 5 studies aimed at finding postpartum dyspareunia among women who experienced perineal injury. Barret et al., in a cross sectional investigation, evaluated dyspareunia combined with major or minor perineal injury, at 3 and 6 months postpartum [37]. Signorello et al. conducted a similar investigation, however it was a retrospective cohort [38]. Mous et al. and Solana-Arellano et al. performed different study design studies (the first is a retrospective cohort study and the second a case control study) on postpartum women at different time span (10 years/2-6 months) and also after distinct degrees of perineal laceration, the first focusing only on anal sphincter injury [39, 40]. Women with major perineal injury endured dyspareunia significantly more frequently and longer than those with minor or no injuries [37–40]. A 4-year-long prospective cohort in UK evaluated women who had had anal sphincter injury (major perineal laceration) at 3 months after childbirth, and described persistent dyspareunia among 32 % of them, but no control group was available for comparison [41].

Female Sexual Function was evaluated through FSFI questionnaire in 4 studies. There were also 2 additional validated questionnaires addressing this particular outcome. Since none of them performed exactly the same procedures, adequate comparison was not feasible [42, 43]. Otero et al. conducted a retrospective cohort that included women evaluated 18 years after anal sphincter injury at childbirth [44]. Compared to women without major injuries, there was no difference regarding FSFI scores below 25 (severe sexual dysfunction). Studies focusing on women with minor or major perineal injury in the postpartum period at 3 days and 6 weeks [45], after 6 years [46] or after 6 and 12 months [47] showed no differences in total FSFI mean scores. The procedures used and time of follow-up in each study were too heterogeneous to allow for direct comparisons and quantitative synthesis.

Additionally, delay to resume sexual activity after childbirth was also a common finding among cohort studies comparing minor and major perineal laceration at delivery. Retrospectively analysed at 6-month post-delivery, it took 2 weeks longer for women who underwent 3^rd^ and/or 4^th^ degree rupture to restart sexual activity, when compared to those with minor lacerations [38]. Prospectively, similar findings were available after 1 year [42], 6 months [48] and 3 months postpartum (and not longer than 6 months) [47]. Through a different overview, a prospective cohort including only women who suffered anal injury at birth showed that 57 % of them were sexually active at 3 months after delivery [41].

Table 3 shows the only 2 prospective cohorts identified that investigated severe maternal morbidity/near miss as exposure [11, 49]. Resumption of sexual activity after childbirth was evaluated at both publications. Nevertheless, they were conducted under different perspectives. Respectively, resumption of intercourse was identified among women exposed and not exposed to severe morbidity after 6 up to almost 8 months after childbirth in the first study, whereas the second one investigated the relationship between perinatal outcome and delay to resume sexual relations at 3, 6 and 12 months postpartum. There were significant differences for the outcome among exposed and not exposed women at both studies. Additionally, Waterstone et al. observed prevalent sexual problems associated with higher depression scores (EPDS) among exposed women [49]. Dyspareunia was evaluated in the study of Ganaba et al., but no difference among exposed and not exposed women was described [11].

Although similarities were found towards exposure and outcomes in the present systematic review, the heterogeneity of studies regarding design, population and type of exposure and outcome measures excluded the feasibility of any quantitative meta-analysis.

## Discussion

Maternal morbidity and maternal near miss have been recently described as health indicators, along with maternal death [1]. Thus, follow up of the women who survived episodes of obstetric complications might provide information for future care of this population. This study aimed to systematically review data available about possible associations of any type of obstetric complication or morbidity during pregnancy, childbirth and extended postpartum period on women’s sexual life aspects, including anatomic changes and/or self-perception of sexual function evaluated through specific tools. Altered female sexual response has already been described as a possible occurrence after pregnancy and delivery [7, 12, 50]. Therefore, it would be reasonable to expect that morbidity during obstetric period would also impact female sexuality. Considering that major perineal injury defined as 3^rd^ or 4^th^ degree lacerations might occur during vaginal births, they could also have influence on postpartum women’s sexual activity.

Surprisingly, this review showed that any type of sexual complaint after childbirth were mostly evaluated in relation to perineal injury as an obstetric complication. In addition, our search did not capture other obstetric morbidities such as hypertensive disorders, postpartum haemorrhage, infection, obstructed labour and even obstetric fistulae as available exposure for analysis in association with possible outcomes related with sexual life and function. Possibly this could represent a limitation of the search strategy used for identifying eligible studies. However, any attempt to include more specific terms for search would probably imply in a huge amount of primarily identified studies which would make the selections process almost impossible. In addition, maybe obstetric fistulae is considered an important outcome, however much more prevalent in under resourced settings and for under privileged population, with some limitations for the assessment of sexual activity and function. Our findings also showed that the majority of studies reporting general obstetric morbidity did not assess the severity of complications at all as possibly associated with the level of sexual dysfunction. Nevertheless, there are two studies addressing this particular outcome among women who experienced episodes of severe maternal morbidity and/or maternal near miss [11, 49].

The qualitative analysis aimed to identify and compare findings of each study regarding similar exposure and outcome, and to perform meta-analysis for a quantitative synthesis when feasible. Nonetheless, the substantial heterogeneity found in the methods used in the included studies, such as different time periods for the evaluation of sexual dysfunction and diverse study designs made any meta-analysis impossible. Still, comparisons among studies suggest that major perineal injury is associated with persistent and longer lasting dyspareunia, in comparison with women with intact perineum and/or minor injuries. The largest difference among groups was observed after 3-month postpartum period. At 6 months after childbirth, only a small part of exposed women was still complaining. These findings have important implications for practice in terms of recommending health professionals that an adequate assistance to the second period of delivery would avoid or decrease perineal injury that is associated with dyspareunia and later resumption of sexual activity.

Comparatively, FSFI scores, both total and below 25 (severe sexual dysfunction) did not differ among exposed and not exposed postpartum women. One study described lower mean scores of both arousal and orgasm domains of FSFI questionnaire on exposed women, without any impact on the total scores [47]. Meanwhile, it is worth mentioning that the mean FSFI scores found on studies included at this review were lower than expected, ranging around 26 among exposed and not exposed women. Despite high probability of arousal dysfunction diagnosis when total scores are below 25, the usual cut off value for suspected altered response is below 26 [51].

Although already validated and widely published, questionnaires addressing female sexual function have several limitations. Women with suspected dysfunction through each of one of these instruments should be individually evaluated for possible diagnosis. Subjective aspects of sexual function and its association with mental health issues, especially depression, might not be distinguished from any score result. Since most questionnaires are self-applied, it might not be feasible to evaluate whether suspected dysfunction is due to physical injury or mental health state.

In addition, time to resume sexual activity after childbirth was comparatively longer among exposed women. The delay to recommence intimate relation after delivery is not considered a real female function or dysfunction parameter. Notwithstanding, women exposed to both perineal major injury and severe maternal morbidity restarted sexual activity later than those without complications. Indeed, distinctive cultural and characteristic practices among specific populations may interfere with sexual abstinence after pregnancy [52]. Notably, morbidity may also play a role on postponement of sexual activity resumption at those contexts, taking into account that several elements might influence this particular behaviour.

Regarding severe maternal morbidity and maternal near miss, beyond delay to resume sexual activity among exposed women, additional findings were higher prevalence of any sexual problems, and correlation of these problems with depressive symptoms [49]. Particularly among women after near miss, the loss of their child was associated with shorter time before planning the following pregnancy [11]. This could possibly seem the opposite of what western societies would expect with regard to postponing sexual activity and therefore a new pregnancy due to mourning for a lost child. Dyspareunia was also investigated. However, there was no statistical difference among postpartum women who experienced near miss or women without obstetric complications [11].

Recently, a new population of women surviving episodes of severe morbidity has surfaced. For each maternal death, 20 to 30 women may experience these conditions. Broadly speaking, concepts of severe maternal morbidity and near miss were only recently defined [1, 2]. Therefore, investigation of possible repercussions on women´s life after maternal morbidity has been only recently and scarcely studied. Specifically, sexual life aspects and/or dysfunction were rarely evaluated among this population.

## Conclusion

Women experiencing maternal morbidity had more frequently dyspareunia and resumed sexual activity later, when compared to women without morbidity. There were no differences in FSFI scores between groups. These results indicate worse outcomes for those experiencing morbidity. New retrospective or prospective studies with standardized procedures evaluating aspects of life and sexual function among women after episodes of severe morbidity might improve understanding of these outcomes. Notably, consequences that may last longer than 6 weeks after childbirth should be examined. Such studies could provide relevant information for clinical follow-up and reproductive planning to this particular growing population of women.

## Abbreviations

FSFI: Female Sexual Function Index
IRS: Intimacy Relationship Scale
MMQ: Maudsley Marital Questionnaire
MeSH: Medical Subject Heading
MNM: Maternal Near Miss
SMM: Severe Maternal Morbidity
SFQ: Sexual Function Questionnaire
WHO: World Health Organization
